# Supplementary material for: Forecasting the onset and course of mental illness with Twitter data
Source: Sci Rep. 2017 Oct 11;7:13006. doi: 10.1038/s41598-017-12961-9 (PMC5636873; doi:10.1038/s41598-017-12961-9)
Supplement: Supplementary file 1 — Supplement [file 41598_2017_12961_MOESM1_ESM.pdf]

Forecasting the onset and course of mental illness with Twitter data

Andrew G. Reece[a*], Andrew J. Reagan[b,c], Katharina L.M. Lix[d],
Peter Sheridan Dodds[b,c], Christopher M. Danforth[b,c*], Ellen J. Langer[a]

Supplementary Information

I. Weekly model output

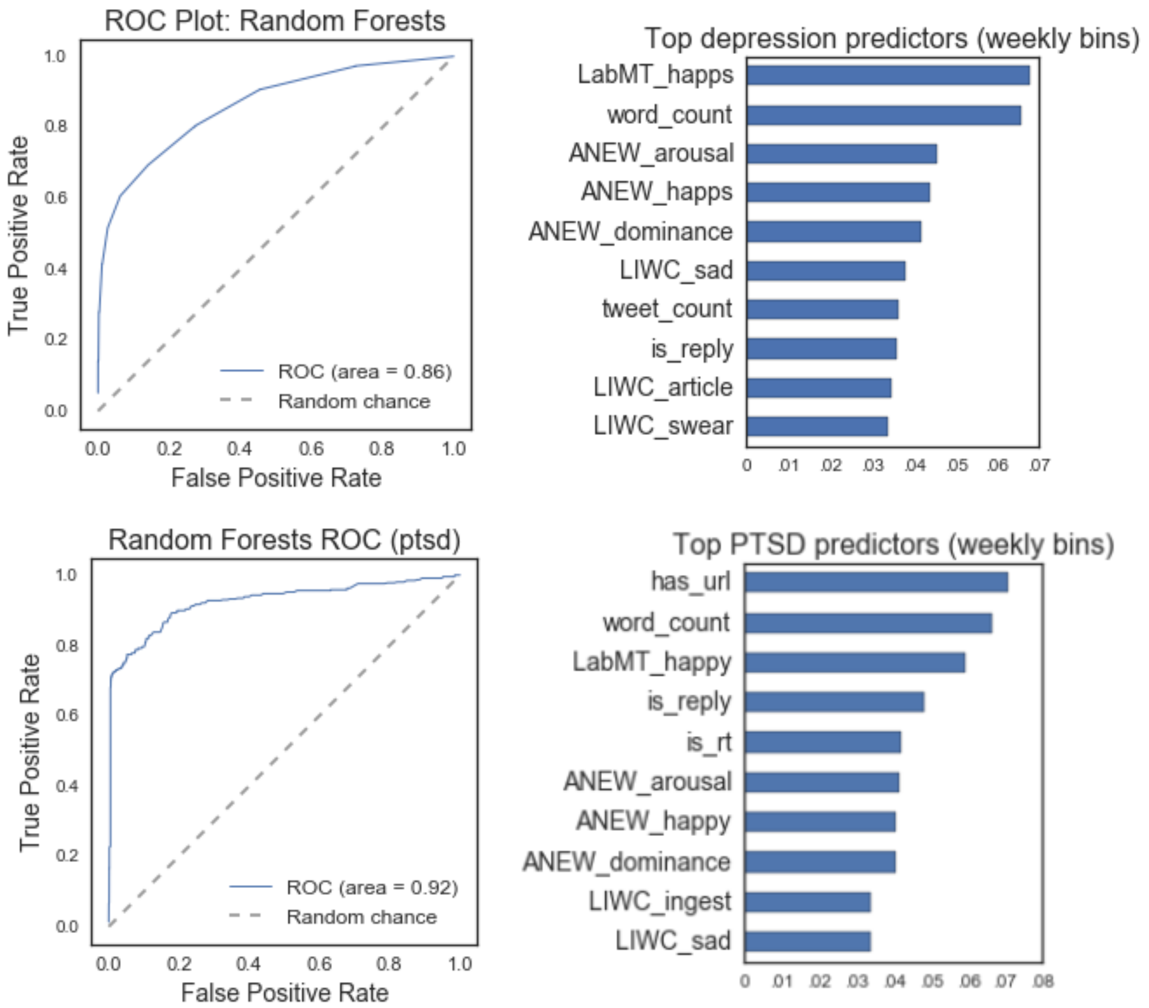

Figure SI 1. ROC curve and top predictors for Random Forests algorithm using weekly units of observation, for depression and PTSD samples (N_{depr}=29,328, N_{ptsd}=12,676). Predictor names ending in "_happy" are happiness measures; LIWC predictors (36) refer to the occurrence of semantic categories (e.g. LIWC_ingest refers to food and eating words, LIWC_swear refers to profanity).

II. Random Forests hyper-parameter optimization

Random Forests parameters were optimized using stratified five-fold cross-validation. The optimization schema is the same as used in Reece and Danforth (12). The optimization routine traversed every combination over the following values (best performing values are highlighted above in red):

n_estimators = [120, 300, 500, 800, 1200]
max_depth = [5, 8, 15, 25, 30, None]
min_samples_split = [1, 2, 5, 10, 15, 100]
min_samples_leaf = [1, 2, 5, 10]
max_features = ['log2', 'sqrt', None]

III. PTSD word shift

#	Word	+/-	↑/↓	% Cont.
1.	photo	+	↑	803.78%
2.	shit	-	↑	-394.46%
3.	no	-	↑	-370.19%
4.	damn	-	↑	-322.25%
5.	theft	-	↑	-312.67%
6.	sucks	-	↑	-291.83%
7.	not	-	↓	283.24%
8.	don't	-	↓	239.32%
9.	love	+	↓	-219.71%
10.	new	+	↓	-216.42%
11.	miss	-	↓	215.76%
12.	hell	-	↑	-214.56%
13.	like	+	↑	209.91%
14.	bad	-	↑	-205.55%
15.	sick	-	↓	166.06%

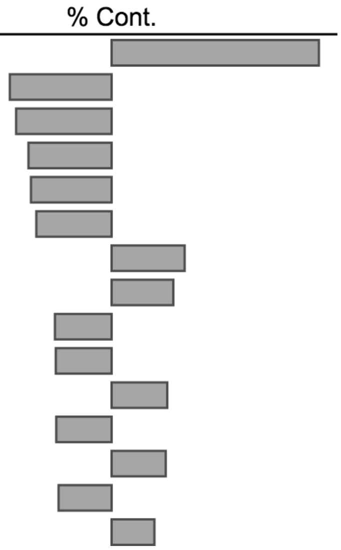

Figure SI 2. PTSD word-shift graph revealing contributions to Twitter happiness observed between PTSD (6.10) and healthy (6.10) participants. In column 3, (-) indicates a relatively negative word, and (+) indicates a relatively positive word, both with respect to the average happiness of all healthy tweets. An up (down) arrow indicates that word was used more (less) by the PTSD class. Words on the left (right) contribute to a decrease (increase) in happiness in the PTSD class. In column 5, % contribution is calculated with respect to the overall average happiness difference between PTSD and healthy participants, which was quite small.
